# Lean back and wait for the alarm? Testing an automated alarm system for nosocomial outbreaks to provide support for infection control professionals

**DOI:** 10.1371/journal.pone.0227955

**Published:** 2020-01-24

**Authors:** Christin Schröder, Luis Alberto Peña Diaz, Anna Maria Rohde, Brar Piening, Seven Johannes Sam Aghdassi, Georg Pilarski, Norbert Thoma, Petra Gastmeier, Rasmus Leistner, Michael Behnke

**Affiliations:** Charité – Universitätsmedizin Berlin, Institute of Hygiene and Environmental Medicine, Berlin, Germany; University of Maryland School of Medicine, UNITED STATES

## Abstract

**Introduction:**

Outbreaks of communicable diseases in hospitals need to be quickly detected in order to enable immediate control. The increasing digitalization of hospital data processing offers potential solutions for automated outbreak detection systems (AODS). Our goal was to assess a newly developed AODS.

**Methods:**

Our AODS was based on the diagnostic results of routine clinical microbiological examinations. The system prospectively counted detections per bacterial pathogen over time for the years 2016 and 2017. The baseline data covers data from 2013–2015. The comparative analysis was based on six different mathematical algorithms (normal/Poisson and score prediction intervals, the early aberration reporting system, negative binomial CUSUMs, and the Farrington algorithm). The clusters automatically detected were then compared with the results of our manual outbreak detection system.

**Results:**

During the analysis period, 14 different hospital outbreaks were detected as a result of conventional manual outbreak detection. Based on the pathogens’ overall incidence, outbreaks were divided into two categories: outbreaks with rarely detected pathogens (sporadic) and outbreaks with often detected pathogens (endemic). For outbreaks with sporadic pathogens, the detection rate of our AODS ranged from 83% to 100%. Every algorithm detected 6 of 7 outbreaks with a sporadic pathogen. The AODS identified outbreaks with an endemic pathogen were at a detection rate of 33% to 100%. For endemic pathogens, the results varied based on the epidemiological characteristics of each outbreak and pathogen.

**Conclusion:**

AODS for hospitals based on routine microbiological data is feasible and can provide relevant benefits for infection control teams. It offers in-time automated notification of suspected pathogen clusters especially for sporadically occurring pathogens. However, outbreaks of endemically detected pathogens need further individual pathogen-specific and setting-specific adjustments.

## Introduction

Outbreak detection of infectious disease is an important area of hospital infection control. It is crucial to detect outbreaks as quickly as possible to limit, through early interventions, the potential for adverse outcomes in affected patients. Technical limitations pose a challenge for establishing a real-time detection system and early recognition of outbreaks [1–3]. In most cases, prospective outbreak detection relies on manual review of pooled microbiological results. This approach is currently being used successfully for rare multidrug-resistant organisms (MDRO)[3]. However, due to the higher number of susceptible organisms in comparison to MDROs, this approach not correlate with the expected outbreak risk in hospitals, but remains widely established as a result of an expected outbreak in hospitals, but remains widely established as a result of the high positive predictive value of very rare pathogens (e.g. with antimicrobial resistance) [4, 5]. The strength of this practical approach lies in the high positive predictive value of very rare pathogens (e.g. with antimicrobial resistance) [4, 5]. When limited human and laboratory resources are taken into consideration, the resulting low number of false positive results makes this outbreak detection manageable.

The increasing digitalization of hospital data offers increasing opportunities for prospective data analyses in modern hospitals [6]. This data can be used to systematically screen for unexpected increases in pathogen detection and thereby make automated outbreak detection systems possible, even for common pathogens [4, 7]. A challenge in finding valuable solutions for automated outbreak detection systems (AODS) is the comparability of the different analytic approaches [3]. In order to assess and compare, a universally agreed on definition of “outbreak” as a gold standard is needed, but is currently still lacking [8, 9].

In this work, our aim was to develop an AODS and to compare its results with our manual approach. Our AODS is based on mathematical methods and the source data was taken from real life hospital data.

## Method

Our AODS is based on regular, computer-based, automated screening and systematic analyses of routinely collected microbiological laboratory and patient location data. In this work, we tested various methods of statistical analysis and compared them to the results of our current manual practice for outbreak detection.

### Databases

The databases derived from real-time diagnostic results of the microbiology laboratory of Charité Universitätsmedizin Berlin. Taken as a whole, the hospital has more than 3,000 beds but is divided into three spatially separated hospital campuses. The individual campuses work mostly independently of each other and exchange patients only irregularly.

The period of the series of new outbreaks investigated in this paper ranged from 01/01/2016 until 12/31/2017. The manual detection of outbreaks occurred prospectively. Each outbreak investigated occurred during this time. The corresponding outbreak records were reported in such a way that the outbreak was at the mid-point of the datasets. Each set contained data for a 12 month period. Thus, it was possible to show data before and after the outbreak. Furthermore, the algorithms utilized by our AODS used historical databases from the same laboratory that cover a 3-year time span from 01/01/2013 and 31/12/2015. The historical databases were necessary to determine the baseline detection frequency of each pathogen. Only in this way could the AODS determine a suspicious change in pathogen detection frequency during the period in question. Data that included the following 11 species was analyzed: the *Acinetobacter baumannii* group (*A*. *baumannii*, *A*. *pittii und A*. *nosocomialis*), *Citrobacter spp*., *Clostridium difficile*, *Enterococcus faecium*, *Enterobacter spp*., *Escherichia coli* (only 3GCREB und CRE), *Klebsiella spp*., *Pseudomonas aeruginosa*, *Salmonella spp*., *Serratia spp*., and *Staphylococcus aureus*. Daily microbiological laboratory data was compiled over a 14-day period. A 14-day period was used because our physicians hold team meetings on a weekly basis to discuss the data from the previous 14 days. This period, which was set by the physicians, is based on transmission time. [10]

These pooled results were defined as a time interval.

The Institutional Review Board ‘Ethikkommission der Charité—Universitätsmedizin Berlin’ waived the requirement for data that is collected in alignment with the German Protection Against Infection Act. The data at hand serves explicitly for infection control purposes within the scope of this regulation.

### Outbreak definitions

#### Established outbreak definition

In order to assess the results of our AODS, we compared the results to those of our manual outbreak detection system. The established system is comprised of pathogen-based daily manual review of bacteriological clinical and screening results as well as of information on infected patients collected by trained infection control physicians on their regular clinical rounds and is focused on MDROs. Whenever a suspicious increase of a certain pathogen is detected, our infection control physicians evaluate the likelihood of an outbreak. This begins with a review of the clinical and epidemiological data on the patients in question. If the likelihood of an outbreak remains high, further microbiological examinations are performed. This includes the collection of clinical microbiological material, the screening of patients in question as well as environmental examinations. A molecular biological assessment is later performed on the pathogens collected for clonality. If this is the case we consider an outbreak to be verified; if not the cluster is considered a false alarm. The outbreak investigation is conducted by at least one infection control resident and is supervised by an infection control attending physician. In addition, several infection control nurses provide basic clinical data and collect the microbiological samples.

#### AODS outbreak definition

**Fig 1:**
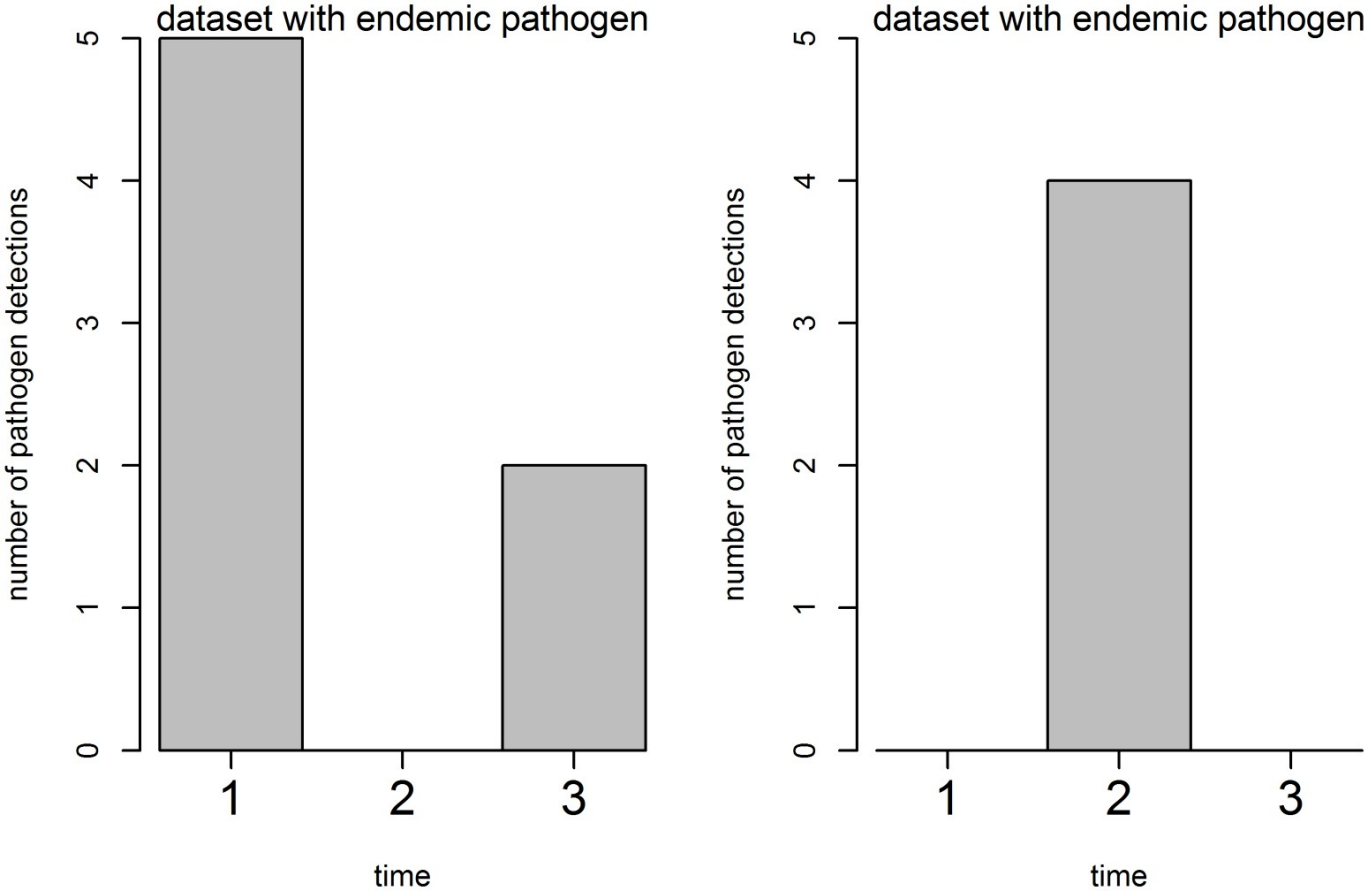
Classification of outbreaks into two types. Datasets with endemically detected pathogens and datasets with sporadic pathogens. In the endemic dataset at least one pathogen occurred more than 30% of the time. In the sporadic dataset at least one pathogen occurred 30% or less of the time.

For the analysis of the AODS, datasets were divided into endemic and sporadic pathogens, based on the frequency of detection per time interval. The criteria for “endemic” was met, when the pathogen was detected in more than 33% of analyzed time intervals. The definition “sporadic”, in contrast, was used for pathogens that were detected in less than 33% of analyzed time intervals. (Fig 1) An aberration was defined as a certain number of pathogens above the endemic level (marked as a colored bar in all figures). An endemic level for each database was determined by the algorithms mentioned above.

In order to develop an AODS with few false positive alarms, we specified a definition for ‘relevant outbreak alarm’ with a high positive predictive value (PPV). Every alarm was considered clinically relevant for datasets of sporadic pathogens. Such pathogens are rarely seen, hence relevant and false positive alarms are rare and no correction for specificity was necessary. Repeated detection of rare pathogens in different patients is, therefore, associated with ad hoc high positive predictive value. In order to ensure a timely alarm, the first (manually detected) outbreak time interval also needed to have triggered an AODS alarm.

Datasets of endemic pathogens are substantially more complicated. They are associated with a high likelihood of false positive alarms which would result in too much work in comparison to the additional benefit for the daily routine. In order to correct for this lack of specificity, we determined that a method is appropriate if 50% or less of the time intervals outside the outbreak were detected as aberrations. This eventually eliminates methods which have too many alarms. In addition, the timeliness of the alarm was ensured by an additional requirement. As with sporadic datasets, the first time interval of the manually detected outbreak needed to have triggered an AODS alarm. For datasets of endemic pathogens, only the combination of these two requirements led to the conclusion. AODS detected the same outbreak as the manual outbreak detection.

### Algorithmic methods

There are five established categories of algorithms used to detect outbreaks. We utilized algorithms from three of these categories: prediction intervals, statistical process control, and statistical modeling. To predict intervals, we used normal distribution prediction intervals (PI-NV), Poisson distribution (PI-POI), and score prediction intervals (PI-SCORE) [11]. As methods of statistical process control, we used the early aberration reporting system (EARS) [12, 13] and negative binomial CUSUMs (NBC) [14]. For statistical modeling, we used the Farrington algorithm [15]. A more detailed description can be found in the supplement.

SatScan [16] based on temporal scan statistics was not used for several reasons. Our system was planned as a daily routine tool for infection control professionals. Furthermore, the technical requirements in our hospital led us to create an intranet application. Therefore, we created a graphical web user interface. At the time we developed our automated outbreak detection system, Satscan did not provide such a solution.

Previous publications have described the feasibility of machine learning for outbreak detection [17]. The method employed by Miller et al. required the measurement of pathogen similarity, e.g. whole genome sequencing. Unfortunately, this data was not routinely available in our hospital during the study period.

All 6 algorithms were used simultaneously. An aberration was detected if the number of isolates exceeded the threshold calculated by the 6 algorithms. Whether an outbreak was identified correctly depended on the pathogen (endemic or sporadic). A detected aberration was considered genuine, if it fulfilled the AODS outbreak definition from the section before. To compare the algorithm we calculated the “detection rate by the algorithms”, i.e. “correct identified outbreaks” divided by “all datasets” multiplied by 100.

All analyses were conducted in *R* [18]. The function “algo.farrington”was used from the package surveillance [19].

## Results

Our infection control team detected 14 outbreaks in the two years analyzed (Table 1 and S1–S12 Figs). Seven outbreaks were found in datasets of pathogens classified as endemic (short endemic datasets), seven in datasets of sporadic pathogens (short sporadic datasets). The 14 outbreaks include six different bacterial species overall. The shortest outbreak duration was a single time interval (14 days), the longest 17 time intervals (238 days). In median, an endemic outbreak lasted 3 time intervals (mean: 6.14 time intervals) and a sporadic outbreak lasted in median 2 time intervals (mean: 2.00 time intervals).

**Table 1:** Overview of manually detected outbreaks in 2016 and 2017. Endemic outbreaks are indicated by Arabic numerals, sporadic outbreaks by Roman numerals.

Outbreak	Pathogen	Drug Resistance	Start Time Interval^1^ of the outbreak	End Time Interval^1^ of the outbreak	Number of Isolates (involved in outbreak)	Time intervals with > = 1 isolates	Type of dataset
1	*Enterococcus faecium*	VRE	9	20	7	22	Endemic^2^
2	*Enterococcus faecium*	VRE	9	18	17	25	Endemic^2^
3	*Staphylococcus aureus*		13	14	6	24	Endemic^2^
4	*Clostridium difficile*		14	14	2	15	Endemic^2^
5	*Clostridium difficile*		14	14	2	15	Endemic^2^
6	*Enterococcus faecium*	VRE	14	16	10	22	Endemic^2^
7	*Enterococcus faecium*	VRE	6	22	9	23	Endemic^2^
I	*Klebsiella* spp	MDR	13	14	3	4	Sporadic^3^
II	*Klebsiella* spp	MDR	14	16	6	6	Sporadic^3^
III	*Escherichia coli*	XDR	13	16	3	3	Sporadic^3^
IV	*Klebsiella* spp		14	14	8	7	Sporadic^3^
V	*Acinetobacter baumannii*	XDR	13	15	3	5	Sporadic^3^
VI	*Clostridium difficile*		14	14	3	7	Sporadic^3^
VII	*Clostridium difficile*		14	14	2	4	Sporadic^3^

VRE, vancomycin-resistant enterococci. MDR, multidrug-resistant. XDR, extensively drug-resistant.

^1^Time interval equals 14 days.

^2^ Eendemic = Isolates found in more than 1/3 of time intervals investigated.

^3^ Ssporadic = Isolates found in 1/3 time intervals or less.

**Fig 2:**
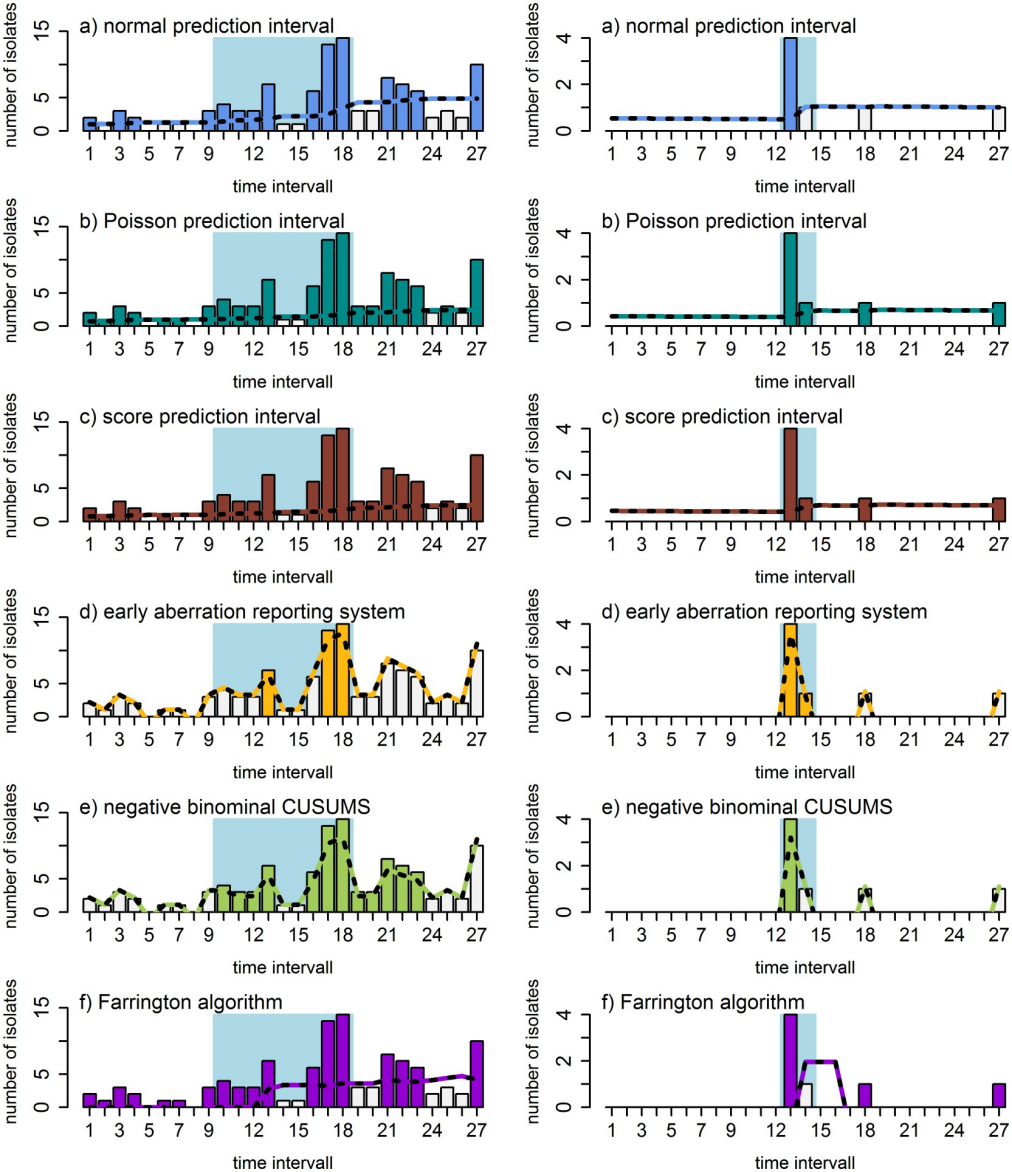
Two examples of outbreaks detected manually vs. outbreaks detected by AODS. Left: Outbreak in an endemic dataset (outbreak 2, vancomycin-resistant *E*. *faecium*). Right: Outbreak in a sporadic dataset (outbreak I, *Klebisella spp*., MDR). Depicted is the course of pathogen detection on the ward during a year when an outbreak was manually detected. The manually detected outbreak is in the center and is indicated by a light blue box. Each bar represents the number of pathogens detected per time interval (14 days). If a bar is colored, an algorithm detected an aberration. Shown are the results for all six algorithms (top down in different colors): normal prediction interval, Poisson prediction interval, score prediction interval, early aberration report system, negative binomial CUSUMs, and the Farrington algorithm.

The respective courses of an endemic and sporadic pathogen are shown in Fig 2. Each bar represents the pathogens detected in one time interval. White bars indicate that no aberration was found, colored bars that an aberration was found. The AODS-calculated thresholds are shown as dotted lines in the figures. The blue boxes show the time frame of the outbreak by the manual method.

For the endemic dataset shown, a pathogen was found in almost every time interval (24 of 27). For the sporadic dataset at least one pathogen occurred in four time intervals.

The first bar in the outbreak of the endemic dataset was detected as an aberration by 5 of 6 algorithms. This met the first criterion for “outbreak was found” for an endemic dataset. For 3 of the 6 algorithms (POI-PI, SCORE-PI and Farrington algorithm) almost all bars outside the blue box indicate an aberration. This violates the second criterion for the outbreak definition of an endemic dataset. Hence, the endemic outbreak was detected by only two algorithms (NBC and NV-PI).

All endemic outbreaks were detected by normal distribution prediction interval (Table 2). Poisson prediction interval, score prediction interval, and negative binomial CUSUMs detected 6 of 7 outbreaks. Early aberration reporting system detected 5 of 7 outbreaks and the Farrington algorithm detected 1 of 7 outbreaks.

**Table 2:** Detection rate for endemic datasets, stratified by results from the 6 algorithms used. The detection rate is shown for each algorithm (columns) and each outbreak (rows).

	Normal dirstribution prediction interval		Poisson dirstribution prediction interval		Score prediction interval		Early aberration reporting system		Negative Binomial Cusums		Farrington		Detection Rate of the outbreak
	FF	L50	FF	L50	FF	L50	FF	L50	FF	L50	FF	L50	
Outbreak 1 (VRE)	X	X	X	X	X	X	X	X	X	X	X		83%
Outbreak 2 (VRE)	X	X	X		X			X	X	X	X		33%
Outbreak 3 (Staphylo-coccus aureus)	X	X	X	X	X	X	X	X	X	X	X	X	100%
Outbreak 4 (CDIF)	X	X	X	X	X	X					X		50%
Outbreak 5 (CDIF)	X	X	X	X	X	X	X	X	X	X	X		83%
Outbreak 6 (VRE)	X	X	X	X	X	X	X	X	X	X	X		83%
Outbreak 7 (VRE)	X	X	X	X	X	X	X	X	X	X			83%
**Detection rate of the alorithms**	**100%**		**86%**		**86%**		**71%**		**86%**		**14%**		

**FF** (First Found), first outbreak time interval detected as aberration. **L50**, ≤50% of the time intervals outside the outbreak were detected as aberration. **X**, the condition FF or L50 was met. An outbreak detection required that both conditions be met. Coloured background indicates that the outbreak was detected by our Automated Outbreak Detection System. **VRE** Vancomycin resitant Enterococci. **CDIF** *Clostridum difficile*.

Almost every first time interval in an endemic outbreak was detected, except in 4 cases. In 9 of 42 cases of endemic outbreak, the algorithms detected too many false alarms. Six of them resulted from use of the Farrington algorithm.

Every algorithm except negative binomial CUSUMs detected all sporadic outbreaks (Table 3).

**Table 3:** Detection rate for sporadic datasets, stratified by results from the 6 algorithms used. The detection rate is shown for each algorithm (columns) and each outbreak (rows).

	Normal dirstribution prediction interval	Poisson dirstribution prediction interval	Score prediction interval	Early aberration reporting system	Negative Binomial Cusums	Farrington	Detection Rate of the outbreak
	FF	FF	FF	FF	FF	FF	
Outbreak I (MDR Klebsiella spp.)	X	X	X	X	X	X	100%
Outbreak II (MDR Klebsiella spp.)	X	X	X	X	X	X	100%
Outbreak III (XDR Escherichia coli)	X	X	X	X		X	83%
Outbreak IV (Klebsiella spp.)	X	X	X	X	X	X	100%
Outbreak V (XDR Acinobacter baumanii)	X	X	X	X		X	83%
Outbreak VI (CDIF)	X	X	X	X	X	X	100%
Outbreak VII (CDIF)	X	X	X	X	X	X	100%
**Detection rate of the alorithms**	100%	100%	100%	100%	71%	100%	

**FF** (First Found), first outbreak time interval was detected as aberration. **L50**, ≤50% of the time intervals outside the outbreak were detected as aberration. **X**, the condition FF was met. Coloured background indicates that the outbreak was detected by our Automated Outbreak Detection System. **MDR** multidrug resistant. **XDR** extensivily drug resistant. **CDIF** clostridium difficile.

## Discussion

When analyzing routine diagnostic microbiological data, automated outbreak detection systems (AODS) offer the means of screening for outbreaks of both common as well as sporadic pathogens[4]. In this work, our aim was to assess an AODS we developed for our hospital. The main approach was to compare our established manual outbreak detection system with the AODS under real-life conditions.

### Sporadic vs. endemic

Based on a rough epidemiological pattern, we defined two types of outbreak analyses: one for pathogens detected sporadically [7, 11] and one for pathogens with high frequency of detection. We termed such pathogens endemic pathogens. These pose a relevant detection problem as they often produce false positive alarms leading to a high risk of pseudo-outbreaks [20]. In contrast, in the case of sporadically detected pathogens, the positive predictive value of an outbreak alarm is very high. If very rare pathogens are detected in multiple patients in a closed space (e.g. particular ward) within a short time (e.g. one month), an epidemiological correlation is highly likely. In this case, no complicated algorithmic analysis is necessary. The benefit of an automated system, then, lies in its immediate alarm. With endemic pathogens, outbreak investigations require close cooperation with the respective clinical department. Microbiological detection of these common pathogens rarely triggers suspicion of an outbreak based on the recurrence of the pathogen. However, it must be assumed that most outbreaks occur with common pathogens. Hence, it is very likely that there is a large reporting bias favoring outbreaks with rare or MDR pathogens [21]. Highly discriminatory molecular analytical methods, e.g. whole genome sequencing, offer a possible future solution since pathogens can be quickly evaluated if suspicious clusters occur [22]. Unfortunately, for the time being these methods are time consuming, expensive, and require specialists trained in bioinformatics. Therefore, they are not currently available as routine diagnostic methods at most hospitals and often are applied only in situations where there exists high risks of further transmission or to patient health.

### Outbreaks of sporadically detected pathogens

All outbreaks with sporadic pathogens were detected by 5 of 6 algorithms. NBC missed an XDR *E*. *coli-* and XDR *A*. *baumannii* outbreak. In order to provide the infection control team with a real benefit in terms of reaction time, our definition of detection required an alarm at the beginning of the outbreak. Even though the XDR *A*. *baumannii* outbreak was eventually detected, it happened only after a significant delay of more than one month (S4 Fig). The XDR *E*. *coli* outbreak was not detected by NBC at all (S2 Fig). In contrast to our results, Watkins et al. showed that NBC provides better detection rates and fewer false alarms than others, e.g. EARS [13]. Another simulation study demonstrated the superiority of NBC to EARS [23]. Those studies, however, tested NBC for natural outbreaks of disease syndromes with a high number of patients, as in natural outbreaks of viral diseases[13]. It is questionable if the circumstances of these studies are can be compared to our setting (hospital ward, bacterial pathogen, low number of cases). Further studies are needed to assess the detection detection rates of these algorithms, in particular NBC, for outbreaks in hospital wards with sporadic bacteria.

### Outbreaks of endemic pathogens

Concerning outbreaks of endemic pathogens, the algorithm’s ‘normal distribution prediction interval’ was superior to all others and detected every outbreak, whereas Farrington performed worst.

Despite Farrington’s detection of 6 of 7 outbreaks, it produced too many false alarms. Our results concerning the Farrington algorithm stand in contrast to the published literature which report good performance by regression models like Farrington, e.g. in public health in France [24]. However, Farrington was designed to adjust for seasonality, which does not apply in our datasets. Moreover, regression models like Farrington do not perform well for small outbreaks (low number of patients) like those in our hospital and in most other hospital settings [25]. Previously it was shown in a simulation study that Farrington has a low sensitivity [26]. Therefore, it is possible that the Farrington algorithm alone should not be the algorithm of choice for regular hospital outbreaks.

Poisson distribution, score distribution prediction interval as well as NBC performed similarly well and showed weaknesses only regarding a VR-*E*. *faecium* outbreak. In comparison, EARS missed one additional outbreak, one of two *C*. *difficile* outbreaks. The VR-*E*. *faecium* outbreak missed occurred during an increase in VR-*E*. *faecium* incidence that was slow overall. This most likely led to an increase in false alarms in Poisson distribution and score distribution. NBC, however, reacted to this increase with fewer false alarms (higher specificity) but triggered the alarm with an unacceptable delay of more than 1.5 months after the outbreak onset.

Moreover, it should be acknowledged that in several respects, outbreaks with VRE are outstanding examples of infection control practice. First, VR-*E*. *faecium* is currently a very common pathogen with significantly increasing incidence [27] in Germany. In our datasets comprise four different outbreaks which represent the highest prevalence (up to 14 different patients per time period). Second, different VRE isolates from hospital clusters are often not distinguishable using commonly available molecular methods [28]. Moreover, because enterococci are ubiquitous bacteria, transmission routes are highly complex and include various modes of introduction between different hospitals and wards, especially oncology wards with continually returning VRE patients [28, 29]. This eventually leads to low positive predictive value for detected aberrations of clusters on oncology wards (or other high endemic settings). This is different particularly in other disciplines with low VRE prevalence. Therefore, adjusted levels of specificity based on ward discipline are required, notably for VRE in Germany, but possibly also for pathogens with similar epidemiological characteristics.

### Outbreaks of Clostridium difficile infections (CDI)

Outbreaks of toxin-producing *C*. *difficile* are another problematic area of automated outbreak detection. *C*. *difficile* is a ubiquitous bacterium; the associated diarrhea (CDI) usually follows extensive antimicrobial therapy [30]. Therefore, simple pathogen detection without clinical verification of diarrhea is most likely not an adequate approach for CDI outbreak detection. Moreover, whole-genome outbreak studies showed that probably only 40% of nosocomial CDI clusters are clonal [31]. The rest are most probably independent events that follow antimicrobial therapy. They can appear as clusters due to ward-specific policies determining which antimicrobial therapies are used. Therefore, on a high endemic ward, the positive predictive value of a CDI outbreak alarm is low. Even more problematic, most laboratories examine factors other than bacterial cultures, for example toxin production. This often renders it impossible to determine post hoc clonal relatedness. We examined four different *C*. *difficile* clusters. Two of them were in sporadic situations and were detected by all algorithms (detection rate 100%). Of the two other endemic clusters, one was detected with an acceptable detection rate of 83% (outbreak 5). The other one (outbreak 4) was detected by only 50% of the algorithms we tested. A noteworthy difference between these outbreaks was the steeper increase in detection rates during outbreak 5 (compared to outbreak 4) and the associated period prior the outbreak. Our results, therefore, showed that for CDI further adjustments need to be made, in order determine the likelihood of a CDI outbreak.

## Limitations

Our AODS approach is pathogen-based and can only detect possible clonal outbreaks. No gold standard for outbreak detection exists because there is no international consensus on the definition of an outbreak [3, 4, 20]. Therefore, specificity could not be calculated for our AODS. Hence, we have based our analysis on the comparison of the outbreak detection system we currently use and the AODS. Our manual outbreak detection system works prospectively but the AODS works retrospectively. This approach could have missed outbreaks. Hence, such outbreaks could be not analyzed by AODS. These Another limitation exists with regard to clonal typing which was not performed routinely. Therefore, it was not possible to detect the potential for better detection rates of the AODS with respect to our manual outbreak detection. Future prospective studies that include state-of-the-art clonal typing methods are necessary in order to assess further benefits of AODS.

## Conclusion

Our work showed that an automated outbreak system for sporadic bacteria in hospitals can work reliably in many cases. It can provide an early warning system and depends only on timely reports of microbiological results. The greatest benefit of such an automated system lies in the automatic alarm for clusters of otherwise rare pathogens, especially in large hospitals. Regarding more common bacteria, the system resulted in a substantial improvement in one hospital’s outbreak detection detection rates. However, the low positive predictive value of those alarms illustrates the need for further adjustments for various other variables. For example, ward and pathogen-specific characteristics need to be taken into consideration in all analyses because they change the predictive value to a great extent. If outbreak isolates are still retrievable, the alarm would lead to further molecular analyses of the isolates’ genetic relatedness. In most cases, this will not be feasible and a decision on the likelihood of a real outbreak remains dependent on the expertise of the hospital epidemiologist. We believe that currently available technology cannot replace an experienced hospital epidemiologist. However, although it needs further development and evaluation in real life situations, our system provides fundamental work toward a system of automated outbreak detection.

## Supporting information

S1 Fig Outbreak within a sporadic dataset (outbreak II). Shown is the course of pathogen detection on the ward during a year when an outbreak was conventionally detected. The conventionally detected outbreak is centered and marked by a light blue box. Every bar stands for the number of pathogens detected per time interval (14 days). If a bar is colored, an algorithm detected an aberration. Shown are the results for all six algorithms (top down in differing colors): normal prediction interval, poison prediction interval, score prediction interval, early aberration report system, negative binomial CUSUMs and Farrington algorithm.
(TIFF)

S2 Fig Outbreak within a sporadic dataset (outbreak III). Shown is the course of pathogen detection on the ward during a year when an outbreak was conventionally detected. The conventionally detected outbreak is centered and marked by a light blue box. Every bar stands for the number of pathogens detected per time interval (14 days). If a bar is colored, an algorithm detected an aberration. Shown are the results for all six algorithms (top down in differing colors): normal prediction interval, poison prediction interval, score prediction interval, early aberration report system, negative binomial CUSUMs and Farrington algorithm.
(TIFF)

S3 Fig Outbreak within a sporadic dataset (outbreak IV). Shown is the course of pathogen detection on the ward during a year when an outbreak was conventionally detected. The conventionally detected outbreak is centered and marked by a light blue box. Every bar stands for the number of pathogens detected per time interval (14 days). If a bar is colored, an algorithm detected an aberration. Shown are the results for all six algorithms (top down in differing colors): normal prediction interval, poison prediction interval, score prediction interval, early aberration report system, negative binomial CUSUMs and Farrington algorithm.
(TIFF)

S4 Fig Outbreak within a sporadic dataset (outbreak V). Shown is the course of pathogen detection on the ward during a year when an outbreak was conventionally detected. The conventionally detected outbreak is centered and marked by a light blue box. Every bar stands for the number of pathogens detected per time interval (14 days). If a bar is colored, an algorithm detected an aberration. Shown are the results for all six algorithms (top down in differing colors): normal prediction interval, poison prediction interval, score prediction interval, early aberration report system, negative binomial CUSUMs and Farrington algorithm.
(TIFF)

S5 Fig Outbreak within a sporadic dataset (outbreak VI). Shown is the course of pathogen detection on the ward during a year when an outbreak was conventionally detected. The conventionally detected outbreak is centered and marked by a light blue box. Every bar stands for the number of pathogens detected per time interval (14 days). If a bar is colored, an algorithm detected an aberration. Shown are the results for all six algorithms (top down in differing colors): normal prediction interval, poison prediction interval, score prediction interval, early aberration report system, negative binomial CUSUMs and Farrington algorithm.
(TIFF)

S6 Fig Outbreak within a sporadic dataset (outbreak VII). Shown is the course of pathogen detection on the ward during a year when an outbreak was conventionally detected. The conventionally detected outbreak is centered and marked by a light blue box. Every bar stands for the number of pathogens detected per time interval (14 days). If a bar is colored, an algorithm detected an aberration. Shown are the results for all six algorithms (top down in differing colors): normal prediction interval, poison prediction interval, score prediction interval, early aberration report system, negative binomial CUSUMs and Farrington algorithm.
(TIFF)

S7 Fig Outbreak within an endemic dataset (outbreak 1). Shown is the course of pathogen detection on the ward during a year when an outbreak was conventionally detected. The conventionally detected outbreak is centered and marked by a light blue box. Every bar stands for the number of pathogens detected per time interval (14 days). If a bar is colored, an algorithm detected an aberration. Shown are the results for all six algorithms (top down in differing colors): normal prediction interval, poison prediction interval, score prediction interval, early aberration report system, negative binomial CUSUMs and Farrington algorithm.
(TIFF)

S8 Fig Outbreak within an endemic dataset (outbreak 3). Shown is the course of pathogen detection on the ward during a year when an outbreak was conventionally detected. The conventionally detected outbreak is centered and marked by a light blue box. Every bar stands for the number of pathogens detected per time interval (14 days). If a bar is colored, an algorithm detected an aberration. Shown are the results for all six algorithms (top down in differing colors): normal prediction interval, poison prediction interval, score prediction interval, early aberration report system, negative binomial CUSUMs and Farrington algorithm.
(TIFF)

S9 Fig Outbreak within an endemic dataset (outbreak 4). Shown is the course of pathogen detection on the ward during a year when an outbreak was conventionally detected. The conventionally detected outbreak is centered and marked by a light blue box. Every bar stands for the number of pathogens detected per time interval (14 days). If a bar is colored, an algorithm detected an aberration. Shown are the results for all six algorithms (top down in differing colors): normal prediction interval, poison prediction interval, score prediction interval, early aberration report system, negative binomial CUSUMs and Farrington algorithm.
(TIFF)

S10 Fig Outbreak within an endemic dataset (outbreak 5). Shown is the course of pathogen detection on the ward during a year when an outbreak was conventionally detected. The conventionally detected outbreak is centered and marked by a light blue box. Every bar stands for the number of pathogens detected per time interval (14 days). If a bar is colored, an algorithm detected an aberration. Shown are the results for all six algorithms (top down in differing colors): normal prediction interval, poison prediction interval, score prediction interval, early aberration report system, negative binomial CUSUMs and Farrington algorithm.
(TIFF)

S11 Fig Outbreak within an endemic dataset (outbreak 6). Shown is the course of pathogen detection on the ward during a year when an outbreak was conventionally detected. The conventionally detected outbreak is centered and marked by a light blue box. Every bar stands for the number of pathogens detected per time interval (14 days). If a bar is colored, an algorithm detected an aberration. Shown are the results for all six algorithms (top down in differing colors): normal prediction interval, poison prediction interval, score prediction interval, early aberration report system, negative binomial CUSUMs and Farrington algorithm.
(TIFF)

S12 Fig Outbreak within an endemic dataset (outbreak 7). Shown is the course of pathogen detection on the ward during a year when an outbreak was conventionally detected. The conventionally detected outbreak is centered and marked by a light blue box. Every bar stands for the number of pathogens detected per time interval (14 days). If a bar is colored, an algorithm detected an aberration. Shown are the results for all six algorithms (top down in differing colors): normal prediction interval, poison prediction interval, score prediction interval, early aberration report system, negative binomial CUSUMs and Farrington algorithm.
(TIFF)

S1 Text Detailed algorithms description. (DOC)

## Abbreviations

Aberration: AODS notification that the number of detected pathogens per time interval is above the expected detection frequency regardless whether an outbreak has been confirmed
Algorithm: Statistical application that evaluates the likelihood of an outbreak behind fluctuating detection frequencies of pathogens based on the comparison to historical data
AODS: Automated outbreak detection system
CDI: *Clostridium difficile* infection
Cluster: An accumulation of phenotypically identical pathogens within a narrow area and time frame
EARS: Early aberration reporting system
NBC: Negative binomial CUSUMs
Outbreak (manual): A cluster of patients with the phenotypically identical pathogen that was considered to derive from nosocomial transmission based on the judgment of the manual outbreak detection system
PI-NV: Normal distribution prediction intervals
PI-POI: Poisson distributed prediction intervals
Time interval: 14 days of microbiological results that were analyzed as a single period
VRE: Vancomycin-resistant enterococci
