# Supplementary figures and images for: Lean back and wait for the alarm? Testing an automated alarm system for nosocomial outbreaks to provide support for infection control professionals
Source: PLoS One. 2020 Jan 24;15(1):e0227955. doi: 10.1371/journal.pone.0227955 (PMC6980399; doi:10.1371/journal.pone.0227955)

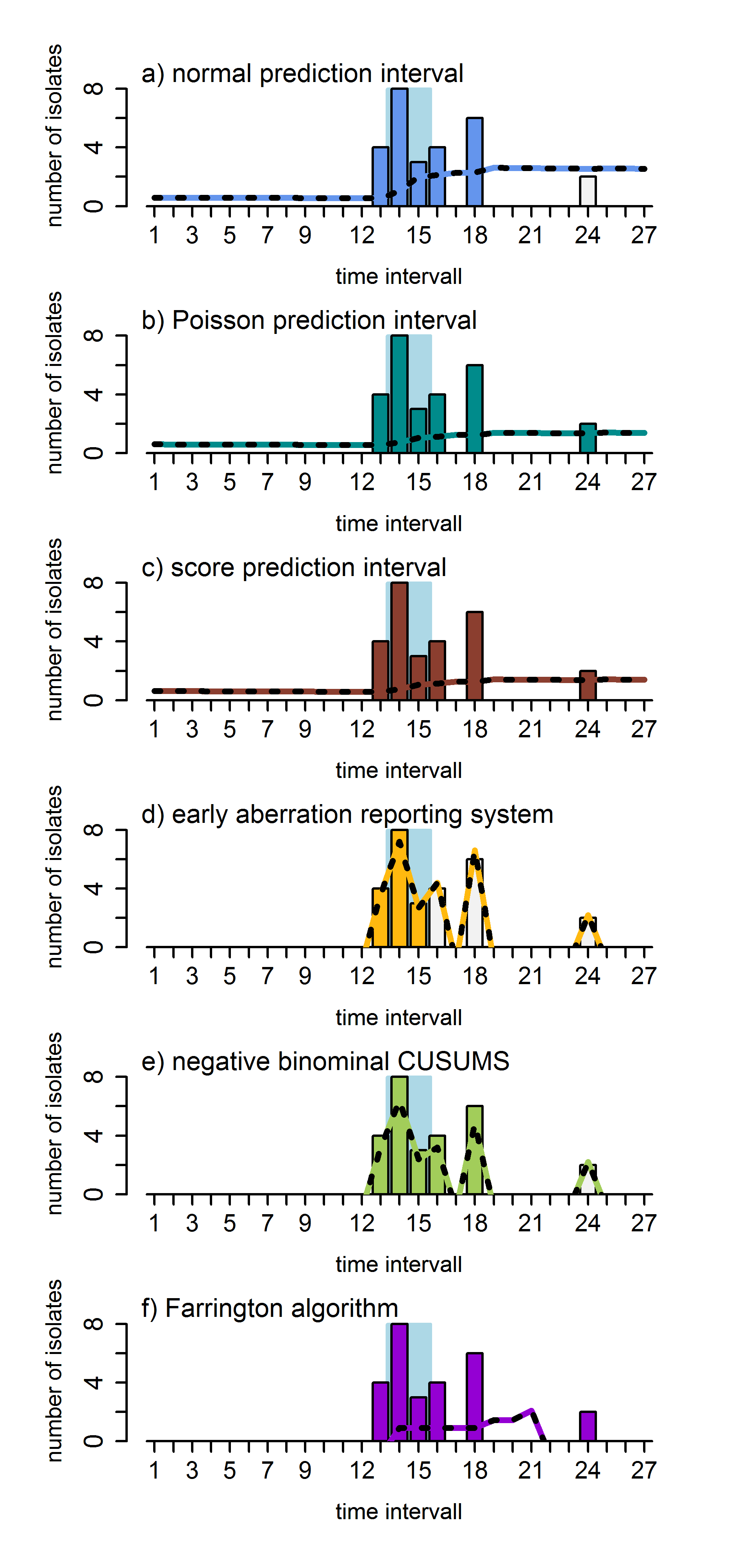

Supplement: S1 Fig — Shown is the course of pathogen detection on the ward during a year when an outbreak was conventionally detected. The conventionally detected outbreak is centered and marked by a light blue box. Every bar stands for the number of pathogens detected per time interval (14 days). If a bar is colored, an algorithm detected an aberration. Shown are the results for all six algorithms (top down in differing colors): normal prediction interval, poison prediction interval, score prediction interval, early aberration report system, negative binomial CUSUMs and Farrington algorithm. (TIFF) [file pone.0227955.s001.tiff]

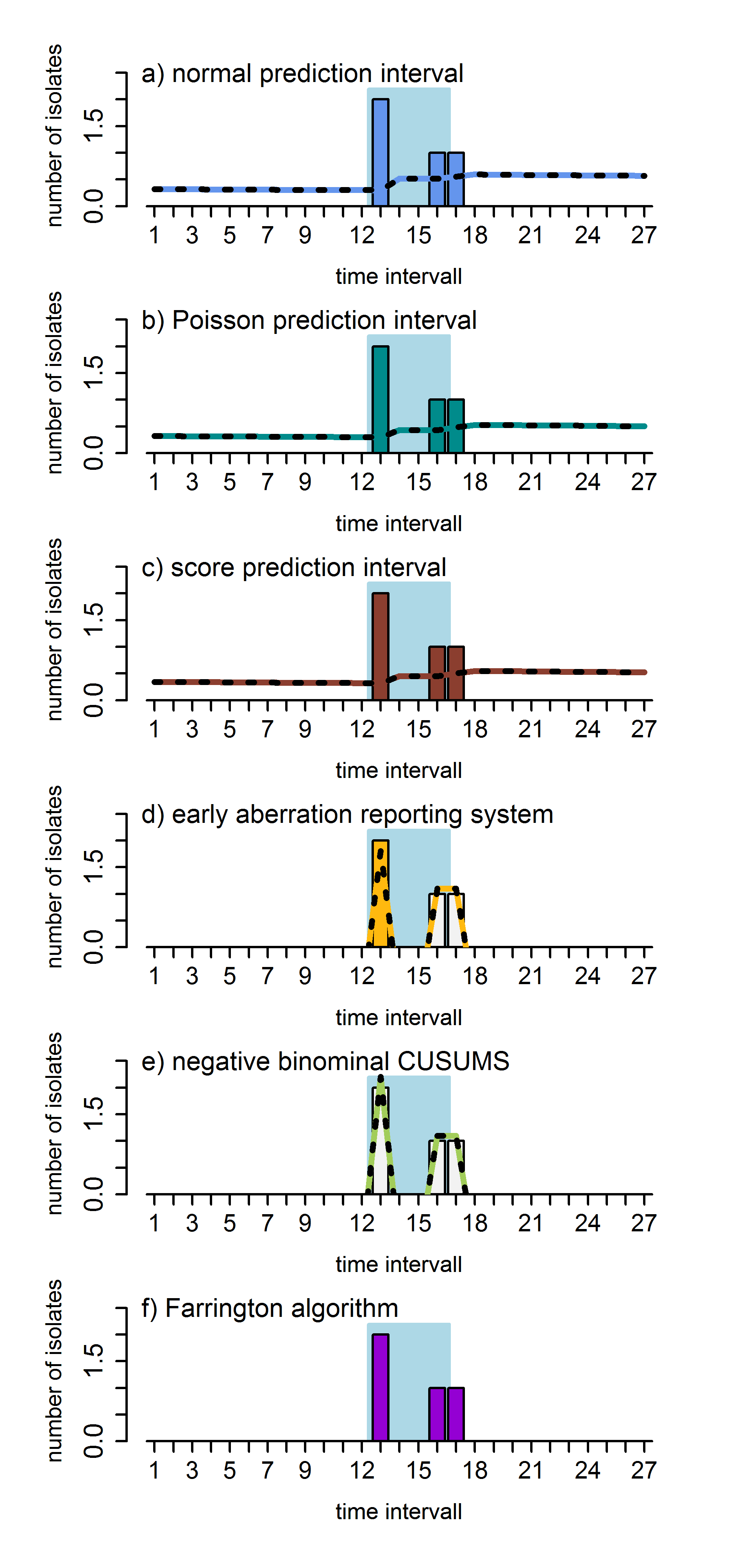

Supplement: S2 Fig — Shown is the course of pathogen detection on the ward during a year when an outbreak was conventionally detected. The conventionally detected outbreak is centered and marked by a light blue box. Every bar stands for the number of pathogens detected per time interval (14 days). If a bar is colored, an algorithm detected an aberration. Shown are the results for all six algorithms (top down in differing colors): normal prediction interval, poison prediction interval, score prediction interval, early aberration report system, negative binomial CUSUMs and Farrington algorithm. (TIFF) [file pone.0227955.s002.tiff]

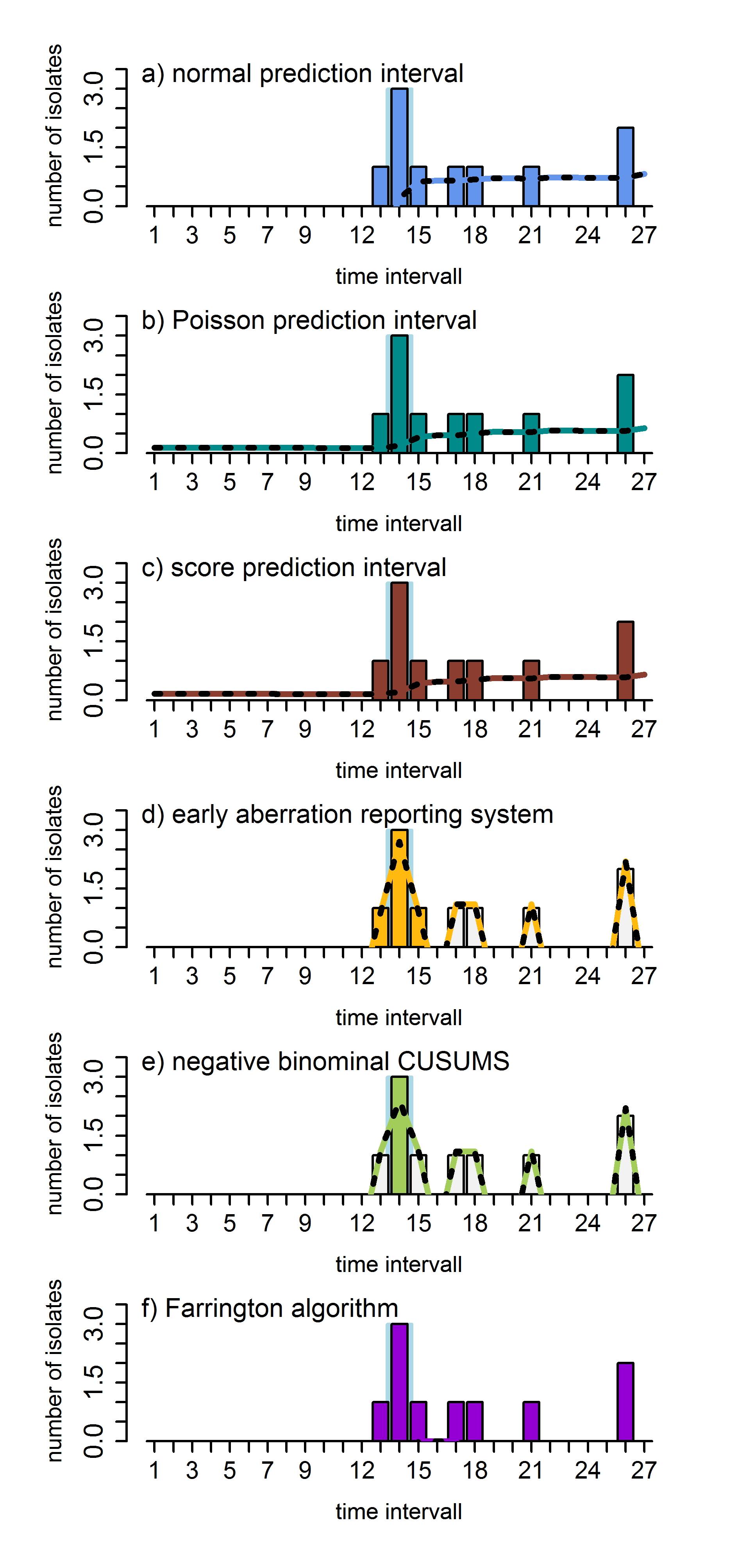

Supplement: S3 Fig — Shown is the course of pathogen detection on the ward during a year when an outbreak was conventionally detected. The conventionally detected outbreak is centered and marked by a light blue box. Every bar stands for the number of pathogens detected per time interval (14 days). If a bar is colored, an algorithm detected an aberration. Shown are the results for all six algorithms (top down in differing colors): normal prediction interval, poison prediction interval, score prediction interval, early aberration report system, negative binomial CUSUMs and Farrington algorithm. (TIFF) [file pone.0227955.s003.tiff]

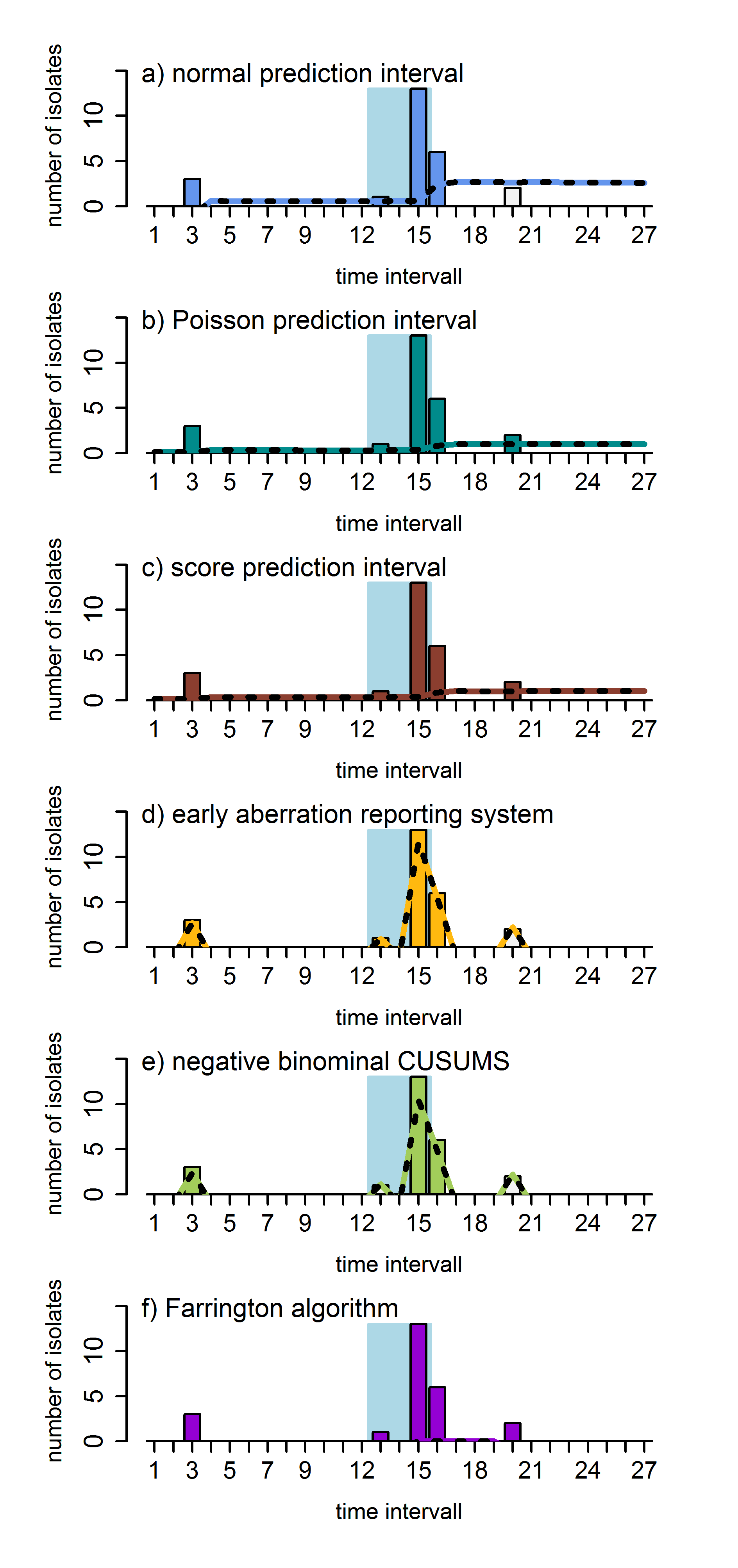

Supplement: S4 Fig — Shown is the course of pathogen detection on the ward during a year when an outbreak was conventionally detected. The conventionally detected outbreak is centered and marked by a light blue box. Every bar stands for the number of pathogens detected per time interval (14 days). If a bar is colored, an algorithm detected an aberration. Shown are the results for all six algorithms (top down in differing colors): normal prediction interval, poison prediction interval, score prediction interval, early aberration report system, negative binomial CUSUMs and Farrington algorithm. (TIFF) [file pone.0227955.s004.tiff]

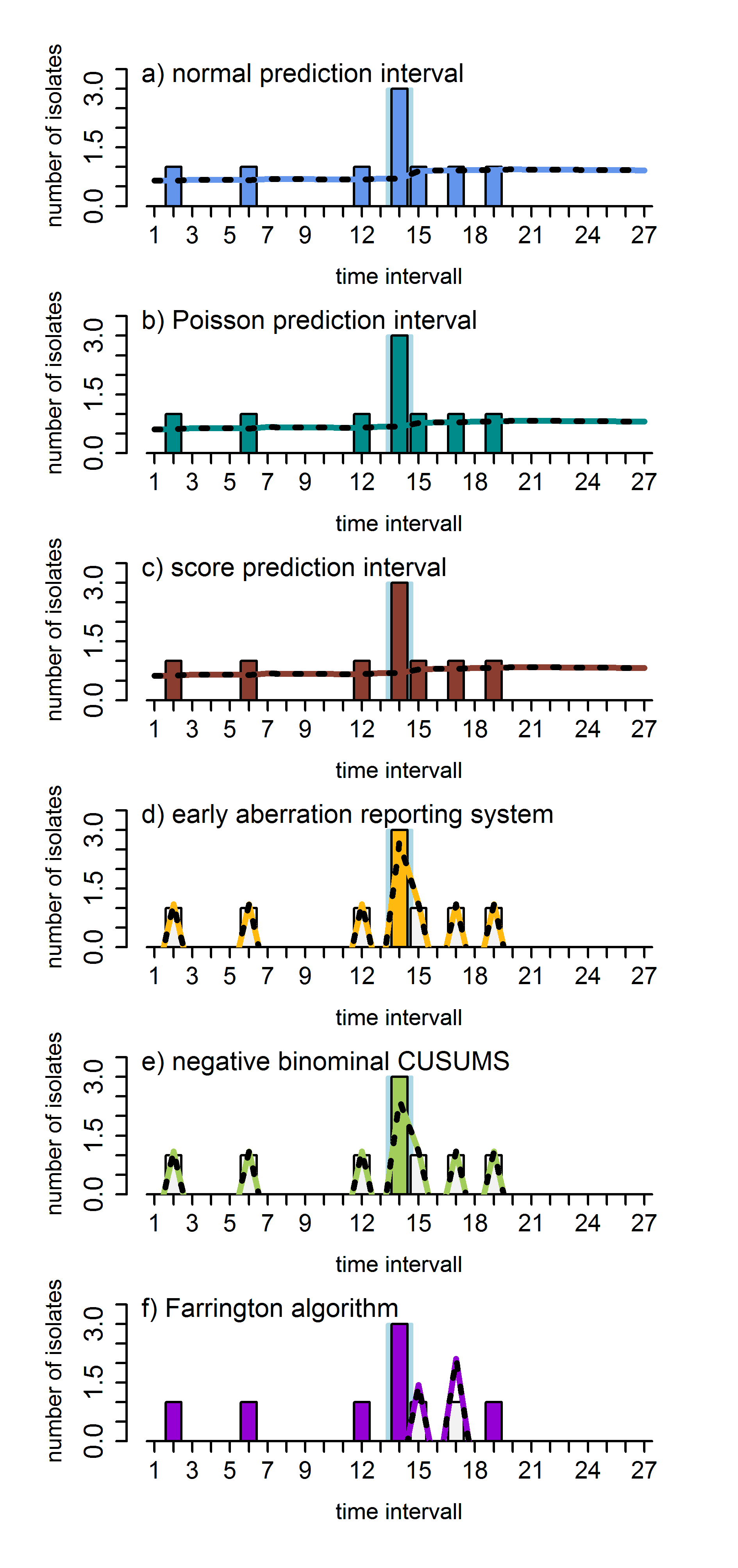

Supplement: S5 Fig — Shown is the course of pathogen detection on the ward during a year when an outbreak was conventionally detected. The conventionally detected outbreak is centered and marked by a light blue box. Every bar stands for the number of pathogens detected per time interval (14 days). If a bar is colored, an algorithm detected an aberration. Shown are the results for all six algorithms (top down in differing colors): normal prediction interval, poison prediction interval, score prediction interval, early aberration report system, negative binomial CUSUMs and Farrington algorithm. (TIFF) [file pone.0227955.s005.tiff]

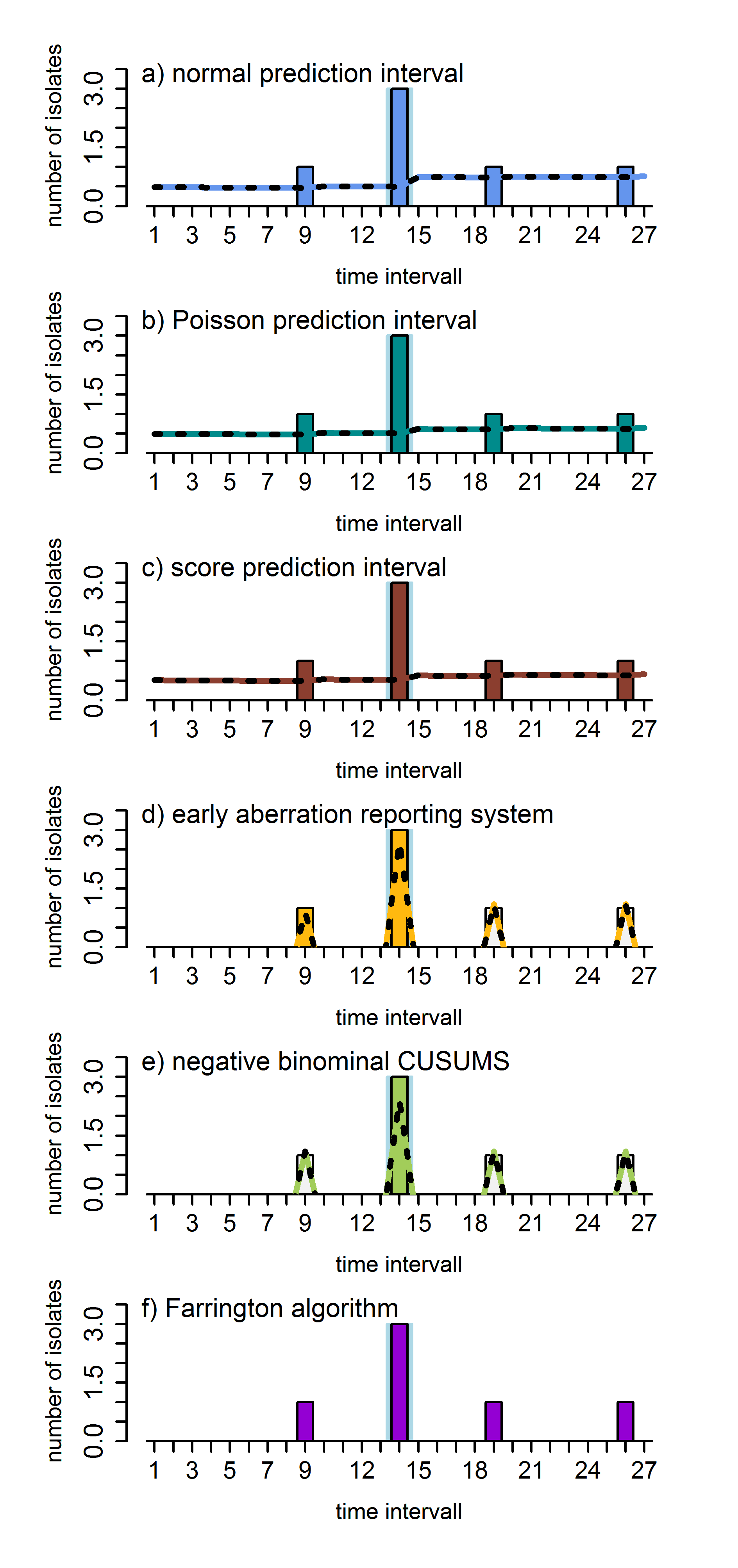

Supplement: S6 Fig — Shown is the course of pathogen detection on the ward during a year when an outbreak was conventionally detected. The conventionally detected outbreak is centered and marked by a light blue box. Every bar stands for the number of pathogens detected per time interval (14 days). If a bar is colored, an algorithm detected an aberration. Shown are the results for all six algorithms (top down in differing colors): normal prediction interval, poison prediction interval, score prediction interval, early aberration report system, negative binomial CUSUMs and Farrington algorithm. (TIFF) [file pone.0227955.s006.tiff]

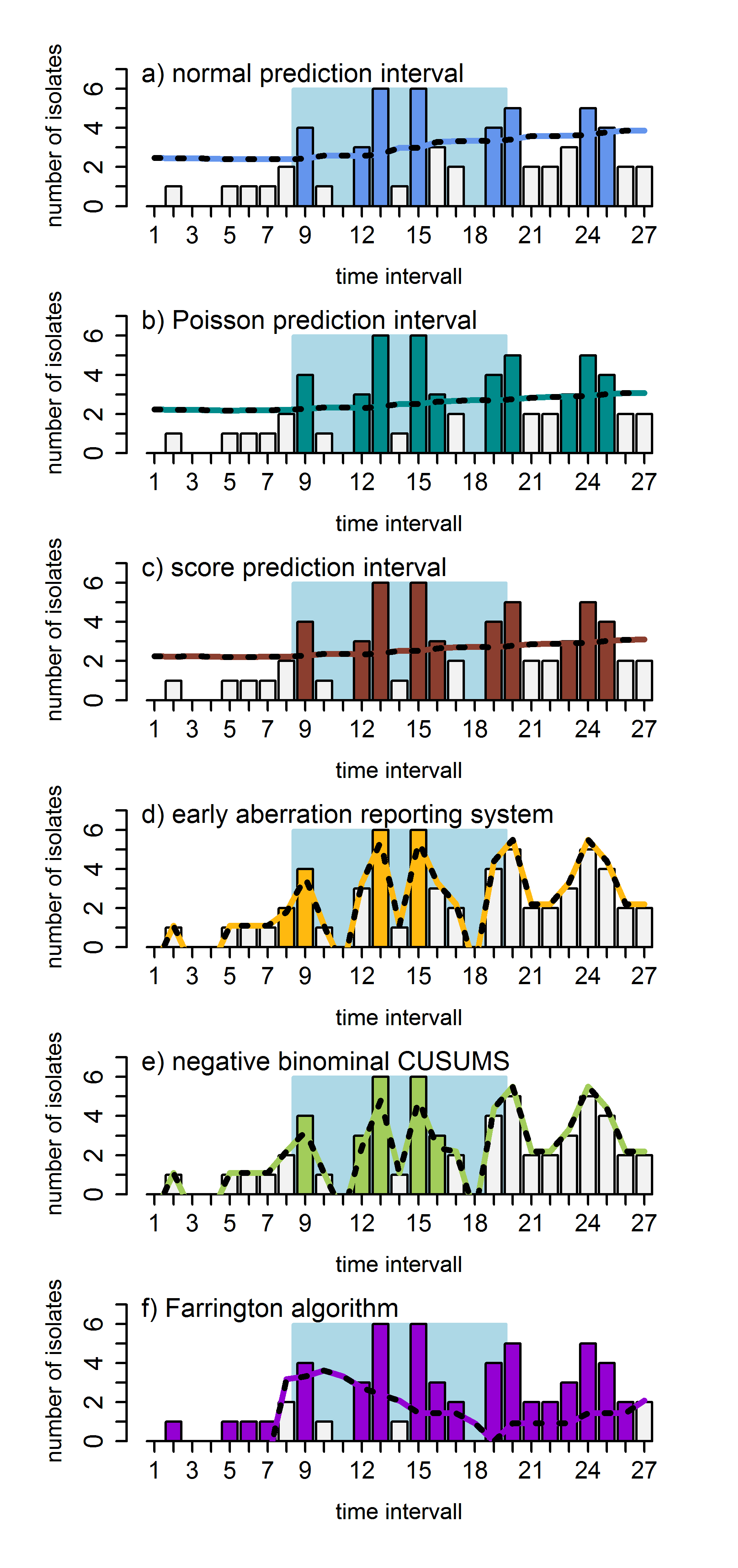

Supplement: S7 Fig — Shown is the course of pathogen detection on the ward during a year when an outbreak was conventionally detected. The conventionally detected outbreak is centered and marked by a light blue box. Every bar stands for the number of pathogens detected per time interval (14 days). If a bar is colored, an algorithm detected an aberration. Shown are the results for all six algorithms (top down in differing colors): normal prediction interval, poison prediction interval, score prediction interval, early aberration report system, negative binomial CUSUMs and Farrington algorithm. (TIFF) [file pone.0227955.s007.tiff]

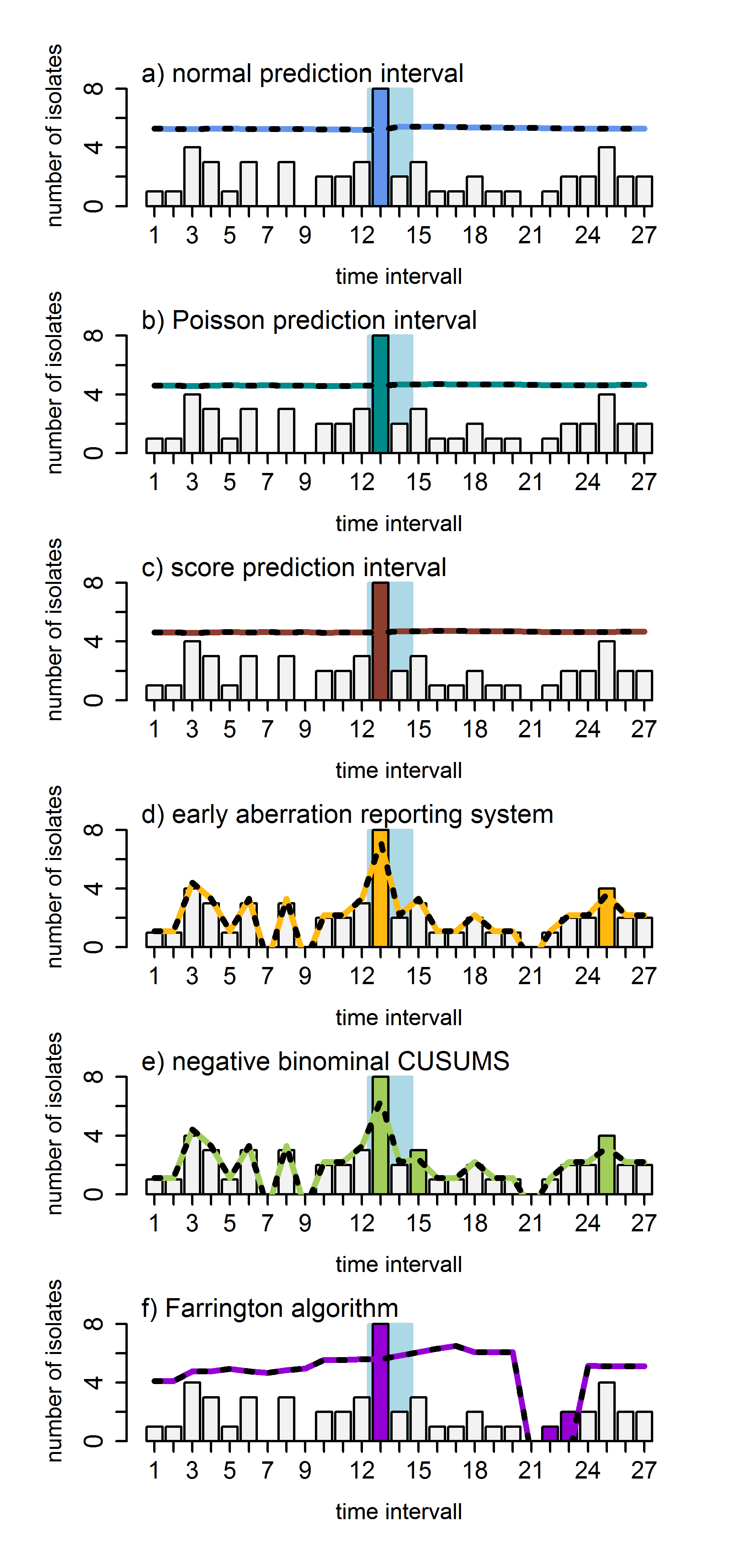

Supplement: S8 Fig — Shown is the course of pathogen detection on the ward during a year when an outbreak was conventionally detected. The conventionally detected outbreak is centered and marked by a light blue box. Every bar stands for the number of pathogens detected per time interval (14 days). If a bar is colored, an algorithm detected an aberration. Shown are the results for all six algorithms (top down in differing colors): normal prediction interval, poison prediction interval, score prediction interval, early aberration report system, negative binomial CUSUMs and Farrington algorithm. (TIFF) [file pone.0227955.s008.tiff]

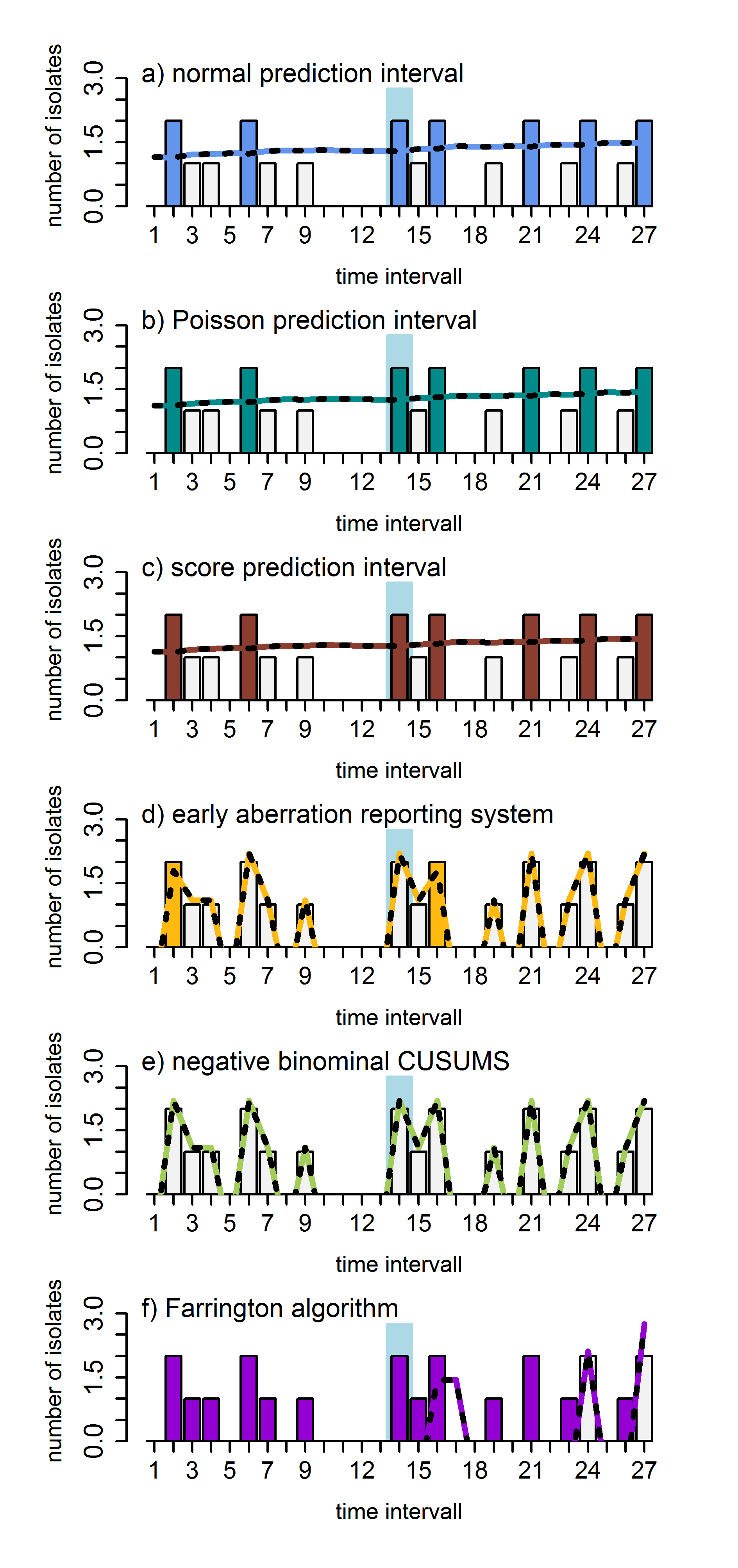

Supplement: S9 Fig — Shown is the course of pathogen detection on the ward during a year when an outbreak was conventionally detected. The conventionally detected outbreak is centered and marked by a light blue box. Every bar stands for the number of pathogens detected per time interval (14 days). If a bar is colored, an algorithm detected an aberration. Shown are the results for all six algorithms (top down in differing colors): normal prediction interval, poison prediction interval, score prediction interval, early aberration report system, negative binomial CUSUMs and Farrington algorithm. (TIFF) [file pone.0227955.s009.tiff]

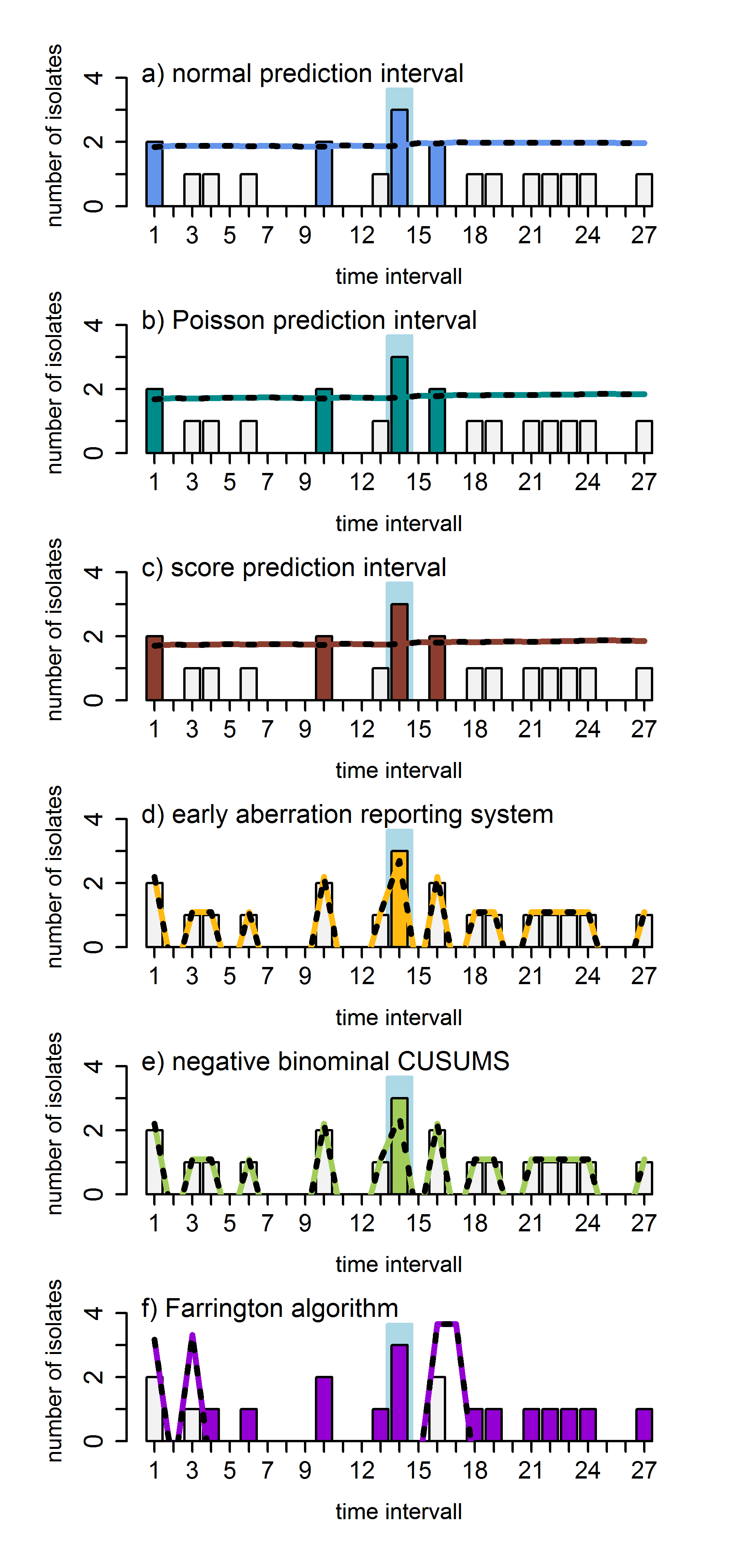

Supplement: S10 Fig — Shown is the course of pathogen detection on the ward during a year when an outbreak was conventionally detected. The conventionally detected outbreak is centered and marked by a light blue box. Every bar stands for the number of pathogens detected per time interval (14 days). If a bar is colored, an algorithm detected an aberration. Shown are the results for all six algorithms (top down in differing colors): normal prediction interval, poison prediction interval, score prediction interval, early aberration report system, negative binomial CUSUMs and Farrington algorithm. (TIFF) [file pone.0227955.s010.tiff]

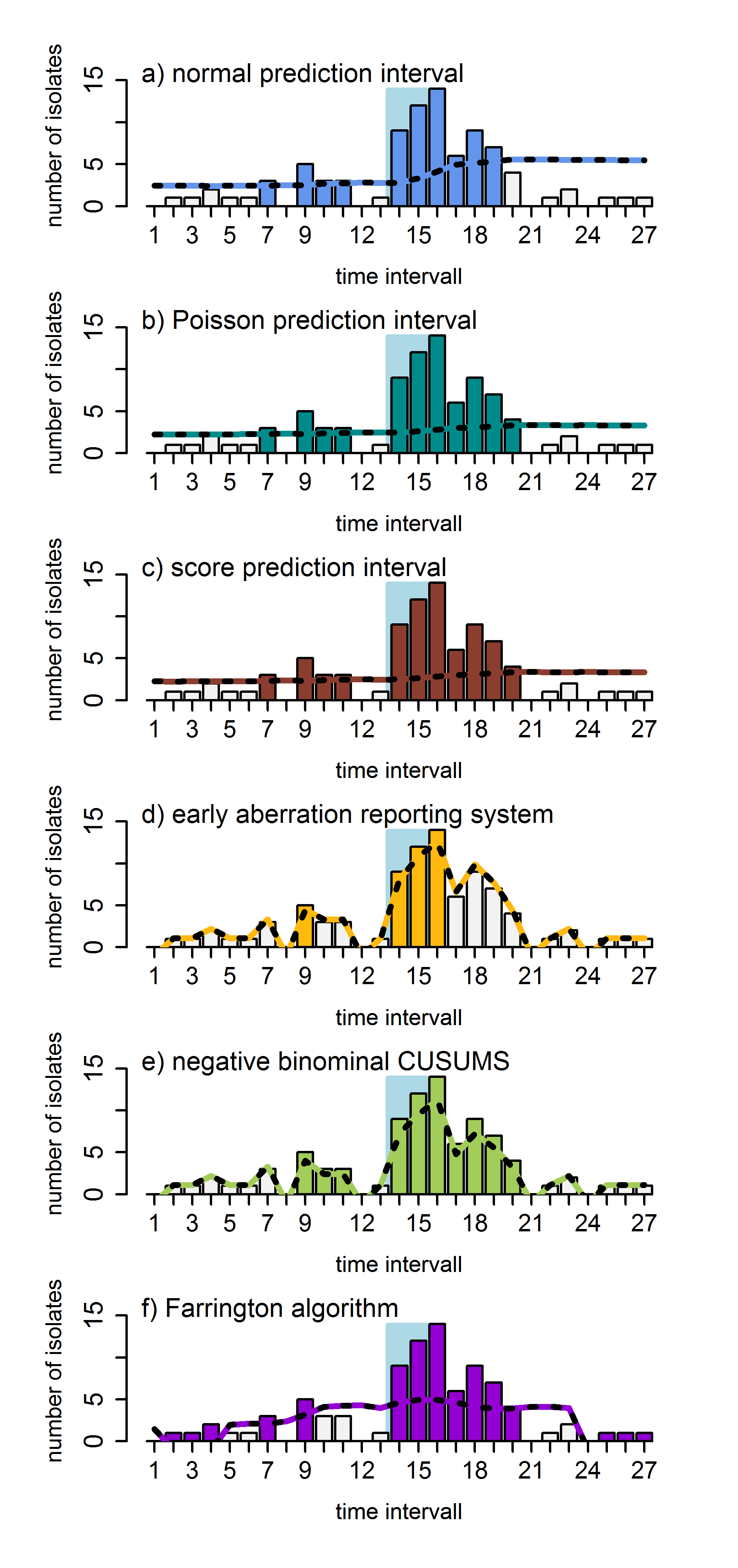

Supplement: S11 Fig — Shown is the course of pathogen detection on the ward during a year when an outbreak was conventionally detected. The conventionally detected outbreak is centered and marked by a light blue box. Every bar stands for the number of pathogens detected per time interval (14 days). If a bar is colored, an algorithm detected an aberration. Shown are the results for all six algorithms (top down in differing colors): normal prediction interval, poison prediction interval, score prediction interval, early aberration report system, negative binomial CUSUMs and Farrington algorithm. (TIFF) [file pone.0227955.s011.tiff]

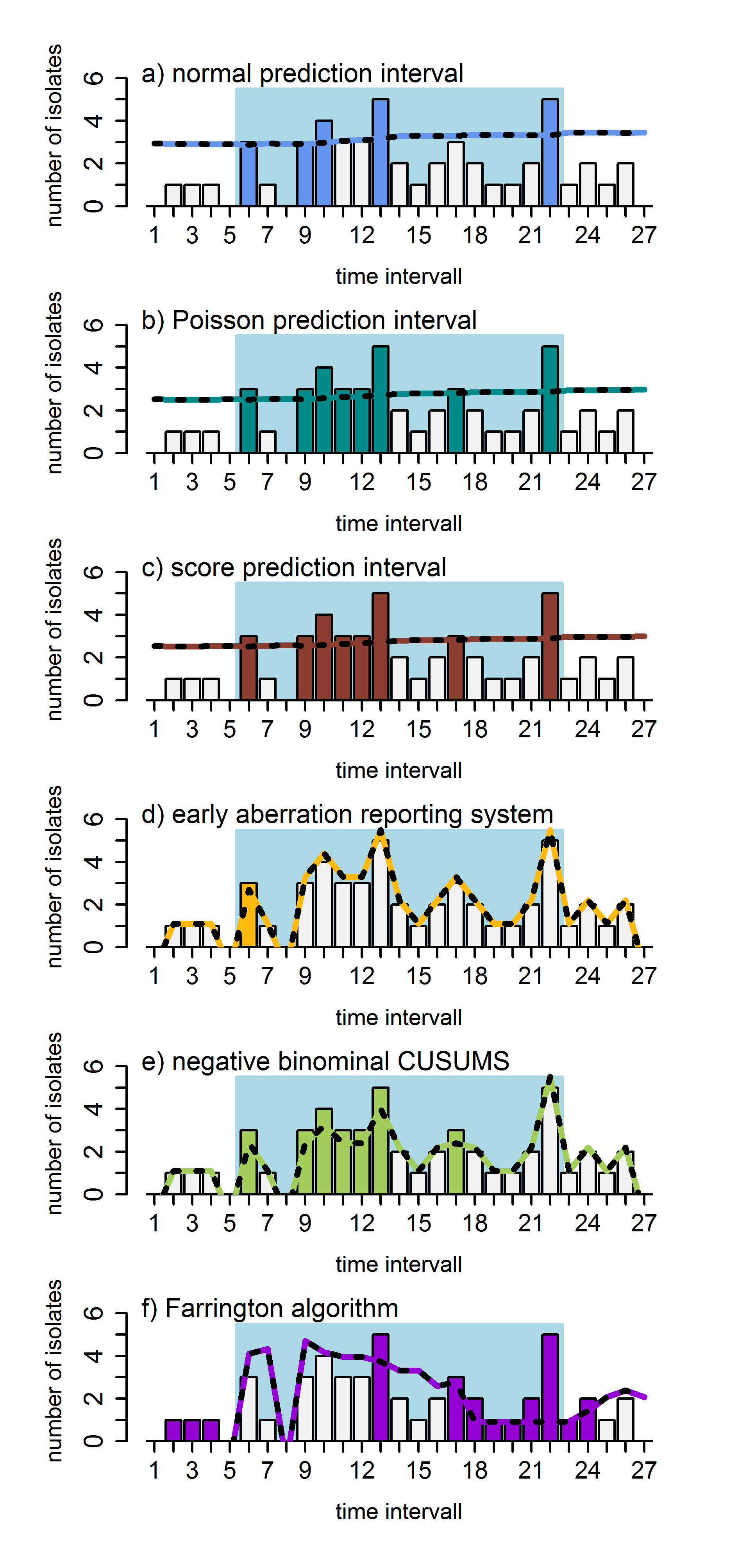

Supplement: S12 Fig — Shown is the course of pathogen detection on the ward during a year when an outbreak was conventionally detected. The conventionally detected outbreak is centered and marked by a light blue box. Every bar stands for the number of pathogens detected per time interval (14 days). If a bar is colored, an algorithm detected an aberration. Shown are the results for all six algorithms (top down in differing colors): normal prediction interval, poison prediction interval, score prediction interval, early aberration report system, negative binomial CUSUMs and Farrington algorithm. (TIFF) [file pone.0227955.s012.tiff]
