# Supplementary material for: Lean back and wait for the alarm? Testing an automated alarm system for nosocomial outbreaks to provide support for infection control professionals
Source: PLoS One. 2020 Jan 24;15(1):e0227955. doi: 10.1371/journal.pone.0227955 (PMC6980399; doi:10.1371/journal.pone.0227955)
Supplement: S1 Text — (DOC) [file pone.0227955.s013.doc]

Prediction Intervals

Calculations were made for three different prediction intervals with different distribution assumptions—a normal distribution prediction interval and a Poisson prediction interval as well as a score prediction interval, which is appropriate for very rare pathogens. [1]

The mean with a corresponding 95% prediction interval was calculated. All data from previous time intervals were used as part of the baseline for the calculation. Thus there are counts for at least 26 time intervals (1 year) which are limits for the count of the current time interval.

Cumulative Sum Control Charts (CUSUMs)

CUSUMs are a method of statistical process control which can be used for public health surveillance. CUSUMS are control charts with memory. A mean is estimated using historical data and a value S is calculated. When S exceeds a threshold value, it indicates an outbreak. We used two different CUSUMs methods. The method from EARS (Early Aberration Reporting System of the Center of Disease Control—a syndromic surveillance system) was one method utilized. The negative binomial CUSUMS method (NBC) was used for some data sets that contain information about rare pathogens which may be not normally distributed or which are over-dispersed. [2-5]

Method C2 [3] was calculated using the EARS algorithm. In other words, 7 previous intervals were used in making the calculation—the 9 time intervals for the current interval with a lag of 2 The same time intervals were used for the calculation of NBC.

Farrington-Algorithm

The Farrington method uses a log-linear regression model adjusted for over-dispersion, seasonality; and trends. [6] Values with high residuals receive a lower weight in regression analyses in order to reduce the influence of outbreaks on the baseline data. The model calculates an expected value for a current time interval based on historical data. The Farrington algorithm needs to have data from at least 1 year because seasonality is analyzed by selecting a baseline comparable to the actual investigated time period, which involves going back to earlier years and looking at four time intervals before and four after the time interval being investigated. Using baseline data from at least 3 years is recommended. It is assumed that an underlying time trend, if one exists, is linear. To be included in the model, data from at least 3 years is needed and the trend has to be significant (p-value < 0.05). It is assumed that underlying data is Poisson distributed. We used 3 years of data. The 2/3 method was used to correct for skewness.

1. Nishiura H. Early detection of nosocomial outbreaks caused by rare pathogens: a case study employing score prediction interval. Osong Public Health Res Perspect. 2012;3(3):121-7.

2. Morton AP, Whitby M, McLaws ML, Dobson A, McElwain S, Looke D, et al. The application of statistical process control charts to the detection and monitoring of hospital-acquired infections. J Qual Clin Pract. 2001;21(4):112-7.

3. Watkins RE, Eagleson S, Veenendaal B, Wright G, Plant AJ. Applying cusum-based methods for the detection of outbreaks of Ross River virus disease in Western Australia. BMC Med Inform Decis Mak. 2008;8:37.

4. Pelecanos AM, Ryan PA, Gatton ML. Outbreak detection algorithms for seasonal disease data: a case study using Ross River virus disease. BMC Med Inform Decis Mak. 2010;10:74.

5. Hutwagner L, Thompson W, Seeman GM, Treadwell T. The bioterrorism preparedness and response Early Aberration Reporting System (EARS). J Urban Health. 2003;80(2 Suppl 1):i89-96.

6. Farrington CP, Andrews NJ, Beale AD, Catchpole MA. A Statistical Algorithm for the Early Detection of Outbreaks of Infectious Disease. Journal of the Royal Statistical Society Series A (Statistics in Society). 1996;159(3):547-63.
